# Genotoxicity of TiO_2_ Nanoparticles in Four Different Human Cell Lines (A549, HEPG2, A172 and SH-SY5Y)

**DOI:** 10.3390/nano10030412

**Published:** 2020-02-27

**Authors:** Fátima Brandão, Natalia Fernández-Bertólez, Fernanda Rosário, Maria João Bessa, Sónia Fraga, Eduardo Pásaro, João Paulo Teixeira, Blanca Laffon, Vanessa Valdiglesias, Carla Costa

**Affiliations:** 1 EPIUnit—Instituto de Saúde Pública, Universidade do Porto, Rua das Taipas, 4050-600 Porto, Portugal; fatimabrandao.988@gmail.com (F.B.); fe.8rosario@gmail.com (F.R.); mjbessa8@gmail.com (M.J.B.); teixeirafraga@hotmail.com (S.F.); cstcosta@gmail.com (C.C.); 2 Environmental Health Department, National Institute of Health, Rua Alexandre Herculano 321, 4000-053 Porto, Portugal; 3 ICBAS—Institute of Biomedical Sciences Abel Salazar, U. Porto—University of Porto, Rua de Jorge Viterbo Ferreira 228, 4050-313 Porto, Portugal; 4 Universidade da Coruña, DICOMOSA Group, Department of Psychology, Area of Psychobiology, Edificio de Servicios Centrales de Investigación, Campus Elviña s/n, 15071 A Coruña, Spain; nataliafb77@gmail.com (N.F.-B.); eduardo.pasaro@udc.es (E.P.); blanca.laffon@udc.es (B.L.); vvaldiglesias@udc.es (V.V.); 5 Universidade da Coruña, Centro de Investigacións Científicas Avanzadas (CICA), Campus Elviña, 15071 A Coruña, Spain

**Keywords:** titanium dioxide nanoparticles (TiO_2_ NPs), uptake, micronuclei, A549, HepG2, A172, SH-SY5Y

## Abstract

Titanium dioxide nanoparticles (TiO_2_ NPs) have a wide variety of applications in many consumer products, including as food additives, increasing the concern about the possible hazards that TiO_2_ NPs may pose to human health. Although most previous studies have focused on the respiratory system, ingestion must also be considered as an important exposure route. Furthermore, after inhalation or ingestion, TiO_2_ NPs can reach several organs, such as the liver, brain or lungs. Taking this into consideration, the present study focuses on the uptake and potential genotoxicity (micronuclei induction) of TiO_2_ NPs on four human cell lines of diverse origin: lung cells (A549), liver cells (HepG2), glial cells (A172) and neurons (SH-SY5Y), using flow cytometry methods. Results showed a concentration-, time- and cell-type- dependent increase in TiO_2_ NPs uptake but no significant induction of micronuclei in any of the tested conditions. Data obtained reinforce the importance of cell model and testing protocols choice for toxicity assessment. However, some questions remain to be answered, namely on the role of cell culture media components on the agglomeration state and mitigation of TiO_2_ NPs toxic effects.

## 1. Introduction

Titanium dioxide (TiO_2_) nanoparticles (NPs) are produced in large scale for use in a wide range of applications [1]. This extensive utilization of TiO_2_ NPs raises questions about their safety in the context of intentional and unintentional human exposure, both occupational and environmental. Most previous in vitro studies investigating TiO_2_ safety have focused on the respiratory system, since inhalation represents the most significant route of exposure to these nanoparticles, mainly in occupational settings [1]. At the environmental setting, personal care products are the largest application for TiO_2_, as these nanoparticles are commonly used in toothpastes for whitening and in sunscreens for protecting skin cells from ultraviolet (UV) light damage [2,3,4]. In addition, oral ingestion is also an important exposure route, particularly because TiO_2_ has been a European Food Safety Authority (EFSA) approved food additive since 1969 (known as E171 coloring food additive) and has been increasingly used in food packaging. After inhalation, dermal exposure, ingestion, or intraperitoneal or intravenous administration (for medical applications), TiO_2_ NPs can reach systemic circulation and be distributed and accumulated in several organs, such as lungs, liver, kidneys, spleen or even brain [1].

As aforementioned, the number of studies on pulmonary toxicity of TiO_2_ NPs outnumbers studies on other target tissues, and in vivo and in vitro data indicate that the main toxicity mechanisms induced by TiO_2_ NPs include inflammation and oxidative stress, as well as genotoxicity [1,5,6,7,8,9,10,11,12]. In rodents, different studies have reported that TiO_2_ NPs can induce lung inflammation and cancer [1]. Based on the evidence of carcinogenicity observed in these studies, TiO_2_ has been classified by the International Agency for Research on Cancer (IARC) as a Group 2B carcinogen “possibly carcinogenic to humans” [13]. In turn, the liver is a major accumulation site for many nanoparticles, including TiO_2_ NPs, due to its physiological and anatomical characteristics [1,14]. These NPs, when deposited in the liver, can cause organ dysfunction, inflammation, DNA oxidative damage, hepatocyte apoptosis and dysregulation of metabolic homeostasis [8,11,15,16,17].

Different in vivo studies have investigated the TiO_2_ NPs biodistribution and demonstrated that these NPs can reach the brain [18,19], either by axonal transport through the olfactory nerve from the nose [20,21], or indirectly, by crossing the blood–brain barrier (reviewed in [19]) or the placental barrier [22]. Because of their slow elimination rates, TiO_2_ NPs may remain in the brain region for a long period, leading to accumulation after repeated exposure [23]. The few studies investigating the neurotoxic effects of TiO_2_ NPs showed that they can induce oxidative stress associated with the production of reactive oxygen species (ROS), apoptosis, autophagy and inflammation, which may lead to cell death and disturb the brain functions, or even induce neurodegenerative disease and psychiatric disorders [18]. Despite the potential mechanisms for TiO_2_ NPs neurotoxicity abovementioned, few studies have focused on the potential genotoxicity of these NPs, and more detailed and standardized research is needed [18,24,25].

Despite the classification of TiO_2_ by IARC in the group 2B [13], its genotoxic potential is still controversial. In fact, results of in vitro genotoxicity studies that have been published are mostly positive, but several negative results have also been reported. According to a recent review on the genotoxic potential of TiO_2_ nanoparticles, seven out of 24 comet assay studies (30%) and four out of 16 micronucleus (MN) test studies (25%) showed no increase in genotoxic damage following exposure to different types of TiO_2_ NPs [26]. Positive findings have been associated with direct interaction with the DNA, after reaching the nucleus [7,27,28,29,30], or indirect mechanisms that include induction of oxidative stress [30,31,32,33,34,35].

Data on the uptake of TiO_2_ NPs by cells are essential to understand their effects and mechanisms. However, contradictory results were observed in studies regarding the TiO_2_ NP uptake behavior by the same cell line or different cell lines [36,37,38]. These contradictory results may be related to factors such as different physicochemical properties of TiO_2_ NPs, their interaction with proteins of biological medium leading to the formation of a protein corona, and chosen cell types, since they have demonstrated to have an important role in TiO_2_ NP uptake and, consequently, in their toxicity [8,36,37,39]. In order to clarify the genotoxic potential of TiO_2_ NPs and how this relates to the cell uptake ability, the present study focuses on the internalization and MN induction of TiO_2_ NPs on four human cell lines of diverse origin: A549 (lung epithelial cell line), HepG2 (liver cell line), A172 (glial cell line) and SH-SY5Y (neuronal cell line). To this aim, the TiO_2_ nanomaterial (Degussa, Evonik, P25 TiO_2_, NM-105, Bitterfeld, Germany), defined as a reference nanomaterial by the Organization for Economic Co-Operation and Development (OECD), was used. Cell lines were exposed to different concentrations of TiO_2_ NPs for two incubation periods: 3 and 24 h.

## 2. Materials and Methods 

### 2.1. Chemicals

Titanium dioxide (TiO_2_ NPs) (CAS No. 13463-67-7) was purchased from Degussa-Evonik (Bitterfeld, Germany). Mitomycin C (MMC) (CAS No. 50-07-7), benzo[a]pyrene (BaP) (CAS No. 50-32-8), 3-(4,5-dimethylthiazol-2-yl)-2,5-diphenyl tetrazolium bromide (MTT) (CAS No. 298-93-1), propidium iodide (PI) (CAS No. 25535-16-4) and Triton X-100 (CAS No. 9002-93-1) were purchased from Sigma-Aldrich Co. (Sintra, Portugal). Ribonuclease A from bovine pancreas (RNAse A) (CAS No. 9001-99-4) was purchased Sigma-Aldrich (St. Louis, MO, USA). MMC, Act-D and BaP were dissolved in sterile distilled water, RNase A was dissolved in ultrapure water, and Cyt-B was dissolved in dimethyl sulfoxide (DMSO) (CAS No. 67-68-5), bought from Sigma-Aldrich Co. (Sintra, Portugal).

### 2.2. Cell Culture

The lung epithelial A549 (ATCC^®^ CCL-185™) cell line was obtained from the American Type Culture Collection (ATCC). Human glioblastoma A172 (ECACC 88062428), hepatocellular carcinoma human cell line HepG2 (ECACC 85011430) and human neuroblastoma SH-SY5Y (ECACC 94030304) cells lines were obtained from the European Collection of Authenticated Cell Cultures (ECACC). A549 was cultured in Dulbecco minimal Eagle’s medium (DMEM) with 1% antibiotic and antimycotic solution (100 U/mL of penicillin, 100 µg/mL of streptomycin and 0.25 µg/mL Gibco Amphotericin B), 1% of Minimum Essential Medium Non-Essential Amino Acids (MEM NEAA) and 10% heat-inactivated fetal bovine serum (FBS). Culture media of the HepG2 and A172 cell lines consisted of DMEM, 1% antibiotic and antimycotic solution and 10% FBS. In turn, the SH-SY5Y cell line was cultured in nutrient mixture EMEM/F12 (1:1) medium with 1% MEM NEAA, 1% antibiotic and antimycotic solution, and supplemented with 10% heat-inactivated FBS. Cells were grown in a humidified atmosphere, with 5% CO_2_, at 37 °C. 

### 2.3. Nanoparticle Suspension: Preparation and Characterization

TiO_2_ NPs were suspended in cell culture media (see composition above), at a final concentration of 200 µg/mL, and ultrasonicated by using a direct tapered microtip (5 mm diameter) attached to a disruptor horn (Branson Sonifier, St. Louis, MO, USA), at 30 W for 5 min (1.5 min on and 1 min off twice, and 2 min on). The TiO_2_ NPs suspension was maintained in ice during the sonication procedure, in order to prevent its heating. Primary size, analyzed by transmission electronic microscopy (TEM), morphology and crystalline phase details were provided by the supplier. In the present study, the hydrodynamic diameter of TiO_2_ NPs, either in deionized water or in A549, HepG2 and A172 complete culture medium, was measured by dynamic light scattering (DLS), using a Malvern Zetasizer Nano ZS (Malvern Instruments, Worcestershire, UK). The zeta potential of TiO_2_ NPs was also evaluated by M3-PALS technique, in the same conditions. 

### 2.4. Exposure to TiO_2_ NPs 

For each assay, different concentrations (10, 50, 100 and 200 µg/mL), chosen according previous studies [8,24,35,36,40], in addition to a negative and a positive control and two exposure periods—3 and 24 h—were evaluated. Culture medium was used as negative control in all experiments, whereas Triton X-100 (1%) in cell culture medium was used as a positive control in cellular viability assessment. For genotoxicity analysis, MMC (1.5 µM for A549 cells and SH-SY5Y and 15 µM for A172), and BaP (80 µM for HepG2) were used as positive controls.

### 2.5. Cellular Viability

Cytotoxicity assessment of TiO_2_ NPs was determined by MTT according to Mosmann [41] for the A172 cell line at the two studied exposure periods (3 and 24 h). These analyses were necessary to guarantee that, at the selected concentrations (10–200 µg/mL), viability was above 60%; for the remaining cell lines, this was confirmed by data available in the literature [24,36,40,42]. Briefly, at the end of the exposure period, the treatment was removed, and 100 μL of MTT reagent, prepared in incomplete medium (0.5 mg/mL), was added to cells and incubated for 4 h, at 37 °C, in the dark. After removing the MTT, the formazan was solubilized with 200 μL of DMSO. To limit potential assay interferences due to the presence of residual NPs, plates were then centrifuged at 1460 *xg*, for 10 min, and 100 μL of the supernatant was transferred to a new plate, for the final reading. Absorbance was measured at 570 nm, with a reference wavelength of 630 nm, using a Cambrex ELx808 microplate reader (Biotek, KC4, Winooski, VT, USA). Potential interaction of NPs with assay components was excluded by a parallel set of experiments conducted without cells. 

### 2.6. Cellular Uptake Evaluation by Flow Cytometry

The potential of the TiO_2_ NPs to enter the cells was evaluated by means of flow cytometry, using a FACSCalibur flow cytometer (Becton Dickinson, Madrid, Spain) and Guava^®^ easyCyte™ flow cytometer (Merck KGaA, Darmstadt, Germany). The analysis was carried out based on the size and the complexity of the cells by measuring the forward scatter (FSC) and the side scatter (SSC), following the protocol described by Suzuki et al. [43] and Zucker and Daniel [44], with some modifications described by [24]. After treatment, cells were washed three times with PBS, to remove the maximum of bound particles, and then detached enzymatically with a 0.25% trypsin-EDTA solution. The content of each well was transferred into a tube and centrifuged for 10 min at 200 *xg*. The supernatant was discarded, and the pellet was gently resuspended in PBS for flow cytometry. SSC signal of 5000 events was analyzed to evaluate the uptake of TiO_2_ by the cells. To calculate the rate of cellular uptake of TiO_2_ NPs (% Cells with NPs), two gates were defined by considering the SSC and the FSC features: one established with control cells (without NPs, R1) and another one positive for SSC (R2, containing cells that have internalized the NPs). The rate of cells containing NPs was calculated as the percentage of R2 cells regarding total cells gated (R1 + R2).

### 2.7. Micronuclei Evaluation by Flow Cytometry

After treatment, cells were cultured for an additional period of 24 h in fresh medium and analyzed for MN frequency as described by Valdiglesias et al. [45]. Briefly, after this additional period, cells were harvested and centrifuged for 5 min at 360 *xg*. The supernatant was removed, and the pellet resuspended in 250 µL of solution I (584 mg/L of sodium chloride, 1 g/L of sodium citrate and 0.3 mL/L of Nonidet P-40), which promotes the disruption of cell membranes, with propidium iodide (PI) (final concentration of 50 µg/mL) and RNase (final concentration of 50 µg/mL). Then cells were incubated for 30 min, at room temperature (in the dark). Subsequently, 250 µL of solution II (15 g/L of citric acid and 0.25 M of sucrose) was added to allow the complete elimination of the cytoplasm, leading to a suspension of nuclei and micronuclei. The cell suspension was then incubated for 15 min, at room temperature, and filtered, using a 55 µm nylon mesh.

### 2.8. Statistical Analysis

A minimum of three independent experiments and two replicates per experiment were performed for each experimental condition tested. Experimental data were expressed as mean ± standard error of the mean. When the distribution of the response variables followed the normal distribution, two-way ANOVA was applied for comparison between group means, followed by Dunnet’s post hoc test; Sidak’s multiple-comparisons test was used to verify possible differences between the two periods of incubation for the same treatment. Nonparametric tests were applied when data distribution significantly deviated from normality; the Kruskal–Wallis test was applied for comparison between groups and Dunn’s multiple-comparisons test, to analyze possible differences between treatments and respective negative control within each period. The Mann–Whitney test was used to verify possible differences between the two periods of incubation for the same treatment. The probability level of 0.05 was used as the criterion of significance. Statistical analyses were performed, using GraphPad Prism 6 for Windows (GraphPad Software, San Diego, CA, USA).

## 3. Results

### 3.1. Nanoparticle Characterization

Table 1 summarizes the physicochemical properties of the TiO_2_ NPs used in the present study. In A549 medium and in HepG2 and A172 medium, TiO_2_ NPs presented a hydrodynamic size of 251.5 and 244 nm, respectively. In turn, a zeta potential of −25.3 mV in deionized water and −11.1 mV, either in A549 medium or HepG2 and A172 medium, was found. Previously performed experiments, following the same protocol of dispersion, showed that the mean hydrodynamic size and zeta potential of these nanoparticles in SH-SY5Y cell culture medium (EMEM/F12) were 228.3 nm and −10.7 mV, respectively (results published in Valdiglesias et al. [24]).

### 3.2. Cellular Viability

**Figure 1:**
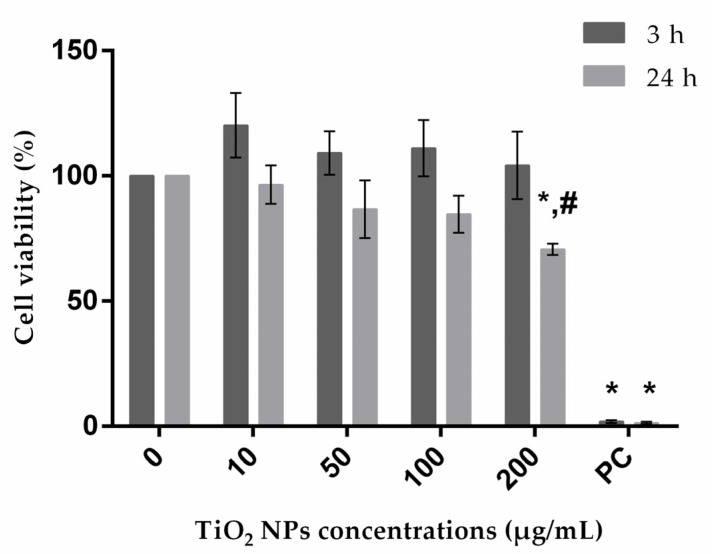
A172 (human glial cell line) viability after treatment with exposure to TiO_2_ NPs evaluated through MTT assay. PC: positive control. * P ≤ 0.05, significant difference in comparison to corresponding negative control, and # P ≤ 0.05 denotes significant difference between the two exposure times (3 and 24 h) (for the same treatment). Data were analyzed by using two-way ANOVA, which was applied for comparison between group means, followed by Dunnet´s post hoc test; Sidak’s multiple-comparisons test was used to verify possible differences between the two periods of incubation for the same treatment.

Cytotoxicity of TiO_2_ NPs on A172 cells was assessed by MTT assay [41]. In this assay, mitochondrial succinic dehydrogenase present in viable cells reduces MTT to water-insoluble blue formazan crystals, which are then solubilized by DMSO; a decrease in signal thus indicates mitochondrial activity impairment. Treatment of A172 cells with TiO_2_ NPs for 3 and 24 h was responsible for a significant decrease in viability only at the highest concentration tested after 24 h of exposure, even though viability was above 60% (Figure 1). For A549, HepG2 and SH-SY5Y cell lines, previous studies had already demonstrated that cell viability is higher than 60% compared to negative control at the range of concentrations used in the current work [24,34,36,40,42].

### 3.3. Uptake Behavior

**Figure 2:**
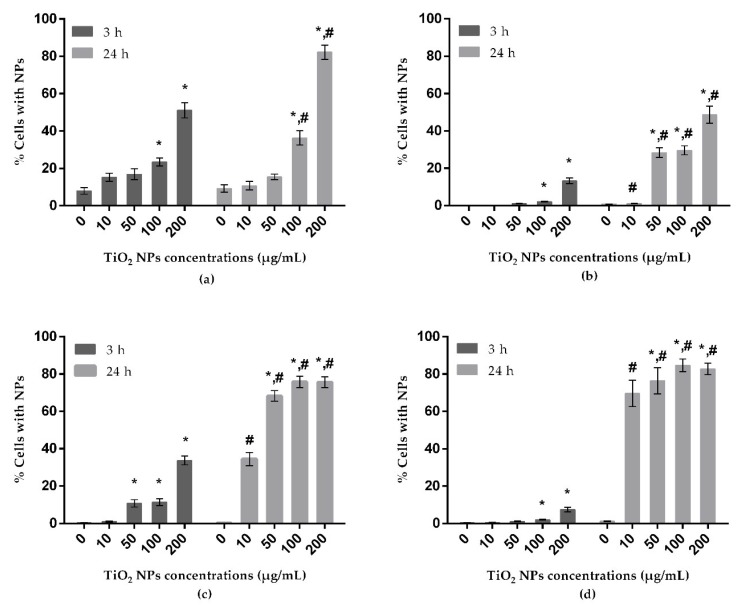
Uptake of TiO_2_ NPs by A549 (human lung epithelial cell line) (**a**), HepG2 (human liver cell line) (**b**), A172 (human glial cell line) (**c**) and SH-SY5Y (human neuronal cell line) (**d**), analyzed by flow cytometry. * P ≤ 0.05, significant difference in comparison to the corresponding control, and # P ≤ 0.05 denotes significant differences between the two exposure times (3 and 24 h) (for the same treatment). Data were analyzed by using the Kruskal–Wallis test for comparison between groups, followed by Dunn´s multiple-comparisons test, to verify possible differences between treatments and respective negative control within each period; the Mann–Whitney test was used to verify possible differences between the two periods of incubation for the same treatment.

Results obtained from uptake analysis are shown in Figure 2. In all tested cell lines, a concentration-dependent increase of uptake of TiO_2_ NPs was observed after 3 h of exposure, but a significant increase was found only at the two highest concentrations tested (100 and 200 µg/mL). In A172 cells, the increase was significant also for the dose of 50 µg/mL. At the longest exposure period, a significant increase of uptake by A549 was observed for the two highest concentrations evaluated. For the remaining cell lines, exposure to TiO_2_ NPs was responsible for significant increases in uptake at lower concentrations: from 50 to 200 µg/mL in A172 and SH-SY5Y cells, and at all doses tested in HepG2 cells. The percentage of uptake in A549 cells was higher comparatively to the same concentrations in HepG2 cells. Moreover, the A172 and SH-SY5Y cells, both cell models of the nervous system, showed a low uptake after the shorter period of exposure (3 h), but after 24 h, they presented the highest levels of TiO_2_ NP uptake. Moreover, a time-dependent increase of NP uptake was noticed in all tested cell lines.

### 3.4. Genotoxicity Assessed with Micronucleus Test

**Figure 3:**
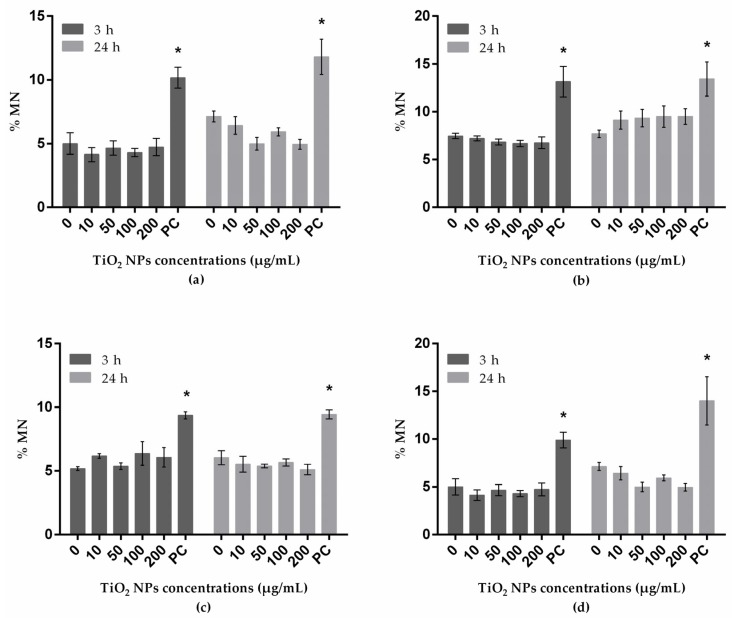
MN frequency determined by flow cytometry in A549 (human lung epithelial cell line) (**a**); HepG2 (human liver cell line) (**b**); A172 (human glial cell line) (**c**); and SH-SY5Y (human neuronal cell line) (**d**) treated with TiO_2_ NPs. PC: positive control. * P ≤ 0.05 significant difference regarding the corresponding negative control. Data were analyzed, using the Kruskal–Wallis test for comparison between groups, followed by Dunn´s multiple-comparisons test, to verify possible differences between treatments and respective negative control within each period; the Mann–Whitney test was used to verify possible differences between the two periods of incubation for the same treatment.

The frequency (%) of MN analyzed by flow cytometry is presented in Figure 3. At the tested conditions, none of the cell lines presented significant increases in MN frequencies.

## 4. Discussion

Previous in vitro studies focusing on TiO_2_ toxicity provide confounding results [26], due to differences in test protocols, different sensitivities of the selected cell models, different cell culture media components and different dispersion techniques used for nanomaterial dispersion [46]. Thus, the objective of this study was to assess the uptake of TiO_2_ NPs (Degussa Aeroxide TiO_2_ NPs P25) and their genotoxic potential in four different cell lines (A549, HepG2, A172 and SH-SH5Y), in a systematic way.

The Degussa Aeroxide TiO_2_ NPs P25 NPs, (also designated as NM-105) have been used in an extensive number of reports on TiO_2_ environmental fate, toxicity and human inhalation [2]. The use of reference nanomaterials for in vitro or in vivo studies is recommended by the OECD, to improve the comparability of interlaboratory test results, even if different biological systems are applied [46]. Dudefoi et al. [47] stated that, even though these NPs are not an optimal model to mimic the nanosized fraction of E171, their use remains of interest, as recent studies have obtained similar effects with both E171 additive and TiO_2_ NPs Aeroxide P25. 

DLS analyses performed by Valdiglesias et al. [24], and in the present study, showed that nanosized particles were successfully obtained in cell culture media. In addition, NP size increased when the NPs were dispersed in media in comparison with water, probably due to the formation of a protein corona. Despite the possible interferences of NP agglomeration and the presence of the corona with endpoints analysis, this is a natural behavior of nanosized particles in biological media, relevant in the assessment of their cellular and molecular effects. 

Data on TiO_2_ NP uptake by cells are essential to understand their effects; internalization may be affected or defined by factors such as the physicochemical properties of TiO_2_ NPs, presence of a protein corona (responsible for alterations in size, charge and agglomeration state), sedimentation, cell cycle phases and cell type [8,36,37,39,48,49]. 

The rapid development of new nanomaterials requires the development of powerful and robust techniques capable of providing data for toxicity assessment, both rapidly and accurately. Thus, TiO_2_ NP uptake was herein investigated by flow cytometry analysis, which allows a faster and more accurate analysis in comparison to other electron microscopy (EM) techniques (e.g., scanning EM [SEM] and transmission EM [TEM]). 

Nanoparticle uptake is largely an active process that is strongly dependent of cell type. Data here obtained demonstrated that A172 and HepG2 cells exposed to a similar TiO_2_ NP suspension present a different pattern of internalization, with A172 cells showing higher levels of internalization in comparison to HepG2 cells for all tested concentrations and exposure periods. This may be due to the presence of a different protein corona, given that its composition also depends on cell proteins, leading to alterations in NP agglomeration state during exposure. In alignment with this, Lankoff et al. [36] observed that the agglomeration state of TiO_2_ NPs had a significant impact on cellular uptake by HepG2 cells. Nevertheless, the biological response of different cell types must not also be disregarded [36]. Furthermore, A549 cells were cultured in a medium very similar to the HepG2 and A172 culture medium, adding only 1% of MEM NEAA, and showed also a different TiO_2_ NPs uptake pattern in comparison to HepG2 (lower uptake than A549) and A172 (higher uptake than A549) cells. Again, this result might be associated with intrinsic cellular characteristics. Similar results were observed by Lankoff et al. [36], who verified that, among three cell lines tested (A549, HepG2 and THP-1), A549 cells were the most active in uptake of all tested particles (Ag NPs and TiO_2_ NPs). 

A172 and SH-SY5Y cells, both cell models of the nervous system, showed a low uptake after the shorter period of exposure (3 h), but after 24 h, they presented the highest % of uptake of TiO_2_ NPs among the four tested cell lines. Valdiglesias et al. [24] also exposed SH-SY5Y cells to the same TiO_2_ NPs used in the current study, following the same dispersion protocol, and found a similar TiO_2_ NPs uptake behavior, demonstrating that results tend to be consistent under similar exposure conditions. In turn, Mao et al. [50] also observed a dose-dependent TiO_2_ uptake by SH-SY5Y, using flow cytometry.

Endocytosis (cellular internalization) and exocytosis (cellular excretion) are active, complex and interdependent processes strongly dependent on cell type [51]. Hence, to explain the lower values of uptake for A549 after 24 h of exposure in comparison to neuronal and glial cells, although they showed the highest % uptake of all cell lines tested after 3 h of exposure, exocytosis of the TiO_2_ NPs cannot be discarded. Furthermore, the equilibrium between internalization and exocytosis is attained at different moments for each cell line, what may explain some of the differences observed for % uptake at both exposure times amongst the four cell lines tested.

The in vitro MN assay is a well-established, reliable, accurate and reproducible endpoint in nano(geno)toxicology, and its use has steadily increased over the last few decades [52]. The traditional assay involves visual scoring, making this a labor-intensive and potentially subjective biased process, limited to two dimensions and limiting the visibility of some MN [53]. On the other hand, MN analysis using flow cytometry is a powerful technique that has the capability of quickly analyzing a huge number of events in three dimensions, leading to a reduction of false negatives [52,53]. Furthermore, the flow-cytometer-based analysis showed a significant correlation with the conventional microscopic based approach [54]. For nanotoxicology, the flow-cytometer-based analysis gives the opportunity to analyze higher doses, limited in the microscopic-based approach due to the difficulties in distinguishing fluorescent particle agglomerates from MN. In line with this, Shukla et al. [27] suggested that the flow cytometry approach was more reliable compared to the microscopy-based conventional method due to the accumulation of particles on slides.

Results here obtained show that, even after a significant internalization of TiO_2_ NP by the four tested cell lines, either after 3 or 24 h, no alterations in the frequency of MN were produced. In accordance, previous studies also observed that the link between internalization and potential toxic effects is not straightforward, as contradictory results have been reported; some studies showed that an efficient TiO_2_ NP internalization is associated with toxic effects [7,10,24,27,30,50,55,56], while others do not observe toxic effects or report low toxicity, even after internalization [36,57]. Previous studies on MN induction with the same cell lines (A549, HepG2 and SH-SY5Y) by TiO_2_ NPs showed inconclusive results. While some of them reported negative results [24,40], in agreement with our results, others observed a significant MN induction [8,9,27,33,34,58]. The use of FBS during NP treatment has been indicated as a possible explanation for negative results, since it was previously reported that proteins present in serum interact with NP surface and reduce cellular responses (e.g., reduction of ROS generation) [59]. However, this is a controversial explanation, since all previous studies that observed MN induction after TiO_2_ NPs exposure have also used FBS during NP treatment. In opposition to what we observed here, Prasad et al. [8] reported MN induction (conventional microscopic-based approach) in HepG2 cells exposed for 24 h to the same NPs in the presence of FBS. In spite of the good agreement previously reported between results obtained by microscopy and flow cytometry methods [34,54], these two methods evaluate the expression of MN in different cell populations. Flow-cytometry method evaluates MN expression in mononucleated cells, while cytokinesis-block MN cytome assay (CBMN) assay discriminates mononucleated cells and once-divided cells (seen as binucleated cells in the presence of cytochalasin-B).

For A172 cells, this is the first report on the potential genotoxicity of TiO_2_ NPs; the few previous studies focused on the effects of TiO_2_ NPs, more specifically Aeroxide TiO_2_ P25 NPs, on glial cells (brain BV2 microglia), and observed an immediate and prolonged release of ROS; upregulation of inflammatory, apoptotic and cell cycling pathways; and downregulation of energy metabolism [55,56].

There are several non-mutually exclusive explanations for the lack of induction of MN after an efficient internalization of TiO_2_ observed herein. In vitro, TiO_2_ NPs have been shown to accumulate in cells mainly by endocytosis and to distribute in intracytoplasmic compartments [31,33,36,50,54,55,56,57]. Some reports also indicated their presence in the nucleus of cells [27,29,60], but in small proportion compared with the observed amount in cytoplasmic compartments, making direct interaction between DNA and TiO_2_ NPs unlikely. Moreover, Xu et al. [61] reported that subcellular localization of nanoparticles plays a critical role in the toxicity profile, with lower toxicity when NPs are localized in the cytoplasm of cells and higher reactivity when nanoparticles are accumulated in nuclei and lysosomes. Furthermore, premutagenic lesions, as the ones detected by the comet assay, may be removed by an efficient DNA repair system before their conversion to mutations or chromosome breaks (i.e., the lesions detected in the MN test). In agreement, Bessa et al. [62] and El Yamani et al. [63] observed that DNA damage induced by TiO_2_ NPs on A549 and HepG2 cells decreased from 3 to 24 h, indicating a potential induction of DNA repair pathways after the first few hours of exposure. 

## 5. Conclusions

In conclusion, our data demonstrate that NP uptake depends on the concentration, time and cell type. Our study also reinforces the importance of cell model and testing protocol choice for toxicity assessment. Considering that TiO_2_ NPs were effectively internalized by all cell lines, but no genotoxicity was associated, current data suggest that TiO_2_ NPs do not affect chromosomal integrity. The approach here presented may be useful in the genotoxicity assessment of other types of NPs, mainly reference materials in cell lines of different origin and representatives of NPs target organs. High-throughput assays can provide faster analysis and robust statistics, and due to the larger number of events measured, they may be essential for screening purposes and prioritizing hazardous materials, a particularly important issue considering the rapid development of engineered nanomaterial (ENM) manufacturing industry, with consequently accelerated incorporation of ENMs into a wide variety of consumer products. However, some questions remain to be answered, such as the role of cell culture media components (not only FBS) on the agglomeration state and mitigation of TiO_2_ NPs toxic effects; and therefore, further complementary genotoxicity assays to confirm these negative results are necessary.

## Figures and Tables

**Table 1:** Characterization of TiO_2_ NPs.

Nanoparticles	Morphology ^1^	Crystalline Phase ^1^	Size (nm) ^1^ (TEM)	Hydrodynamic Diameter (nm) (DLS)				Zeta Potential (mV) (DLS)			
Degussa, Aeroxide TiO_2_ P25 NPs	Spherical	80% anatase 20% rutile	25	Deionized Water	A549 medium	HepG2 and A172 medium	SH-SY5Y medium	Deionized Water	A549 medium	HepG2 and A172 medium	SH-SY5Y medium
				205.1	251.5	244	228.3 [24]	−25.3	−11.1	−11.1	−10.7 [24]

^1^ Provided by the manufacturer, TEM: transmission electronic microscopy, DLS: dynamic light scattering.
